# CORRIGENDUM

**DOI:** 10.1002/cam4.1887

**Published:** 2018-12-27

The oncogenic roles of nuclear receptor coactivator 1 in human esophageal carcinoma

Lu Wang, Weiwei Li, Kai Li, Yi Guo, Ditian Liu, Zhimeng Yao, Xianjie Lin, Shujun Li, Zuojie Jiang, Qing Liu, Yi Jiang, Beien Zhang, Lei Chen, Fuyou Zhou, Hongzheng Ren, Danxia Lin, Dianzheng Zhang, Sai‐Ching Jim Yeung & Hao Zhang^1^

Cancer Medicine 2018; 7: 5205‐5216

In the published version of this article, the affiliations of Professor Hao Zhang are incorrect and should be modified as follows:

Hao Zhang^12,13^

^12^Department of Pathology, Institute of Precision Cancer Medicine and Pathology, Jinan University Medical College, Guangzhou, China

^13^Department of Immunotherapy and Tumor Tissue Bank, Affiliated Cancer Hospital of Shantou University Medical College, Shantou, Guangdong, China

The authors apologize for this error.
